# *Ausichicrinites zelenskyyi* gen. et sp. nov., a first nearly complete feather star (Crinoidea) from the Upper Jurassic of Africa

**DOI:** 10.1098/rsos.220345

**Published:** 2022-07-20

**Authors:** Mariusz A. Salamon, Sreepat Jain, Tomasz Brachaniec, Piotr Duda, Bartosz J. Płachno, Przemysław Gorzelak

**Affiliations:** ^1^ Institute of Earth Sciences, University of Silesia in Katowice, Będzińska Street 60, 41-200 Sosnowiec, Poland; ^2^ Department of Geology, School of Applied Natural Science, Adama Science and Technology University, 1888 Adama, Oromia, Ethiopia; ^3^ Faculty of Science and Technology, University of Silesia in Katowice, Będzińska Street 39, 41-200 Sosnowiec, Poland; ^4^ Institute of Botany, Faculty of Biology, Department of Plant Cytology and Embriology, Jagiellonian University in Kraków, Gronostajowa Street 9, 30-387 Kraków, Poland; ^5^ Institute of Paleobiology, Polish Academy of Sciences, Twarda 51/55, 00-818 Warszawa, Poland

**Keywords:** crinoids, comatulids, predation, tithonian, Ethiopia, Africa

## Abstract

Fossil comatulids, referred to as feather stars, are mostly known from highly disarticulated specimens. A single isolated element (centrodorsal) has been the basis for taxonomic description of a vast majority of fossil comatulids. Here, we report a nearly complete, and thus extremely rare, comatulid from the Upper Jurassic (Tithonian) of the Blue Nile Basin in central western Ethiopia that provides a unique insight into the morphology of comatulid arms and cirri. It is assigned to *Ausichicrinites zelenskyyi* gen. et sp. nov. and is the first Jurassic comatulid from the African continent. The new taxon shows some similarities with representatives of the Mesozoic Solanocrinitidae but also has close resemblance with the modern family Zygometridae, exclusively known from the Holocene of western Pacific and eastern Indian Oceans. This morphologic similarity is considered to be due to convergence. The first example of pinnule regeneration in a fossil feather star is reported, which reinforces the hypothesis about the importance of predation in the evolution of these crinoids.

## 1. Introduction

Comatulids are the most diversified lineage of Recent crinoids (comprising of *ca* 140 genera) [1,2]. According to Hess & Messing [3] the order Comatulida A.H. Clark comprises three suborders (stalkless as adults—Comatulidina A.H. Clark; and two groups that retain their stalk as adults—Bourgueticrinina Sieverts-Doreck; and Guillecrinina Mironov and Sorokina).

Comatulids, commonly known as feather stars, shed their stalks during ontogeny and display high mobility (through crawling and swimming), which is regarded as a significant factor related to their success [4,5]. They are also the only extant crinoid group that is globally distributed in both shallow- and deep-water settings [6,7].

The fossil record of comatulids dates back to the Late Triassic [8–11]. Of the known comatulid genera, most of the described records are based on a single and the most durable morphological element, the centrodorsal (and are rarely based on centrodorsal with attached basals and radials). This choice of a single ossicle is due to the fact that the comatulids are susceptible to post-mortem processes and thus, disarticulate very rapidly [12].

The centrodorsal, that serves as the interface between the cirri and arms, constituted a major innovation in crinoid evolution. This element is quite morphologically diverse and instantly recognizable as a comatulid. It evolved from a coalesced series of a few cirriferous columnals [9] that arose during ontogeny from a single proximal columnal [13]. Its importance in the taxonomy of fossil comatulids has been long recognized [14]. This is especially true for the Early-Middle Jurassic comatulids, for which morphological disparity of centrodorsals rapidly increased [9]. Despite subsequent diversification during the Cenozoic, the variety of centrodorsal form seems to be saturated, i.e. the evolution proceeded on a variety of designs that were established earlier [2]. Indeed, it has been shown that some distantly related species of Recent comatulids have very similar centrodorsal shapes. Likewise, intraspecific variation in the morphology of the centrodorsal of Recent comatulids may be very high [15]. Hence, neontologists generally do not use centrodorsal characteristic, which is usually the only available parameter for palaeontologists. For the taxonomic description of living comatulids, the characters of the whole animal are taken into consideration, with special emphasis on the morphology of arms and cirri. In this paper, we describe an exceedingly rare find of a nearly complete fossil comatulid that also enabled a unique comparison between fossil and Recent taxa.

## 2. Geological setting and palaeoenvironment

**Figure 1.**
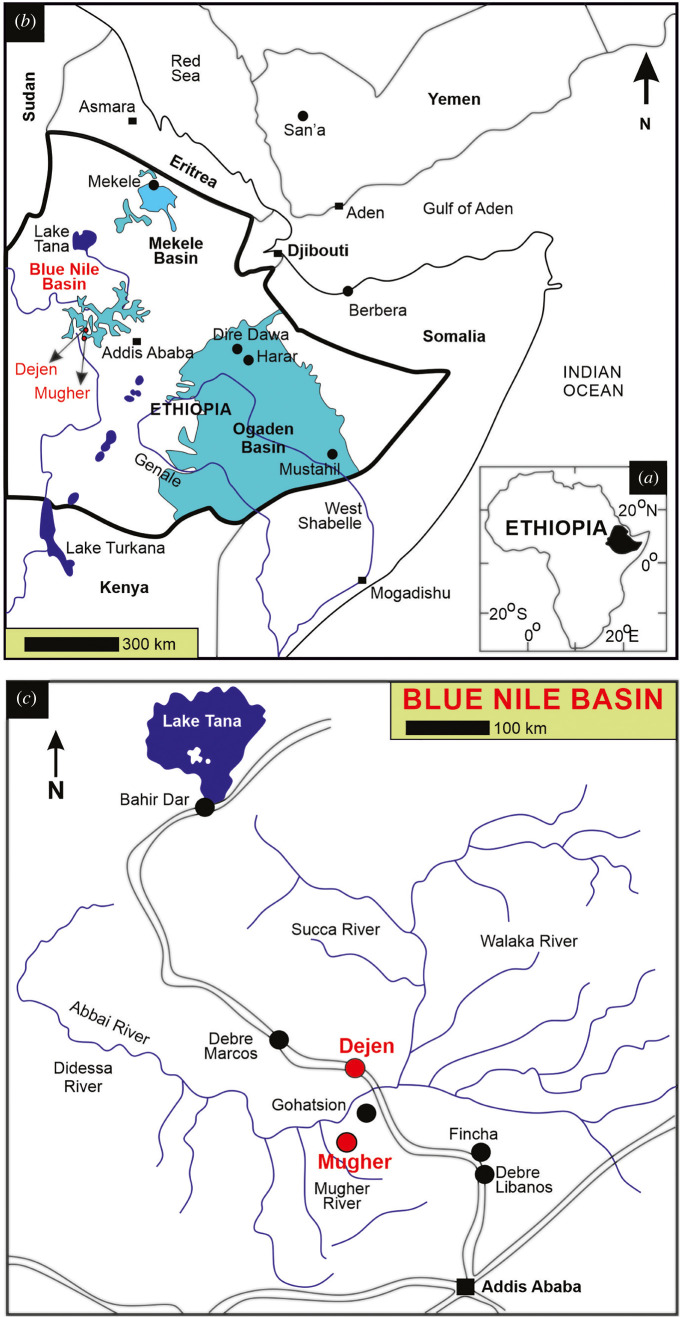
Geological and locality map. (*a*) Geological map of Ethiopia showing the three sedimentary basins, Ogaden, Blue Nile and Mekele (redrawn and slightly modified after [23]). (*b*) Major Jurassic localities mentioned in the text from Ethiopia, Somalia and Yemen. The shaded part in (*b*) (within Ethiopia) is enlarged in (*c*). (*c*) Locality map of the measured section at Mugher and the reference section at Dejen for the Blue Nile Basin (mentioned in the text). (*b*) and (*c*) are redrawn and modified after Jain [20].

The Jurassic sedimentary rocks in Ethiopia are exposed in three basins—Mekele in the north [16–18], Blue Nile in the centre [19–23] and Ogaden along the eastern margin of the Ethiopian Rift [24] (figure 1*a*).

**Figure 2.**
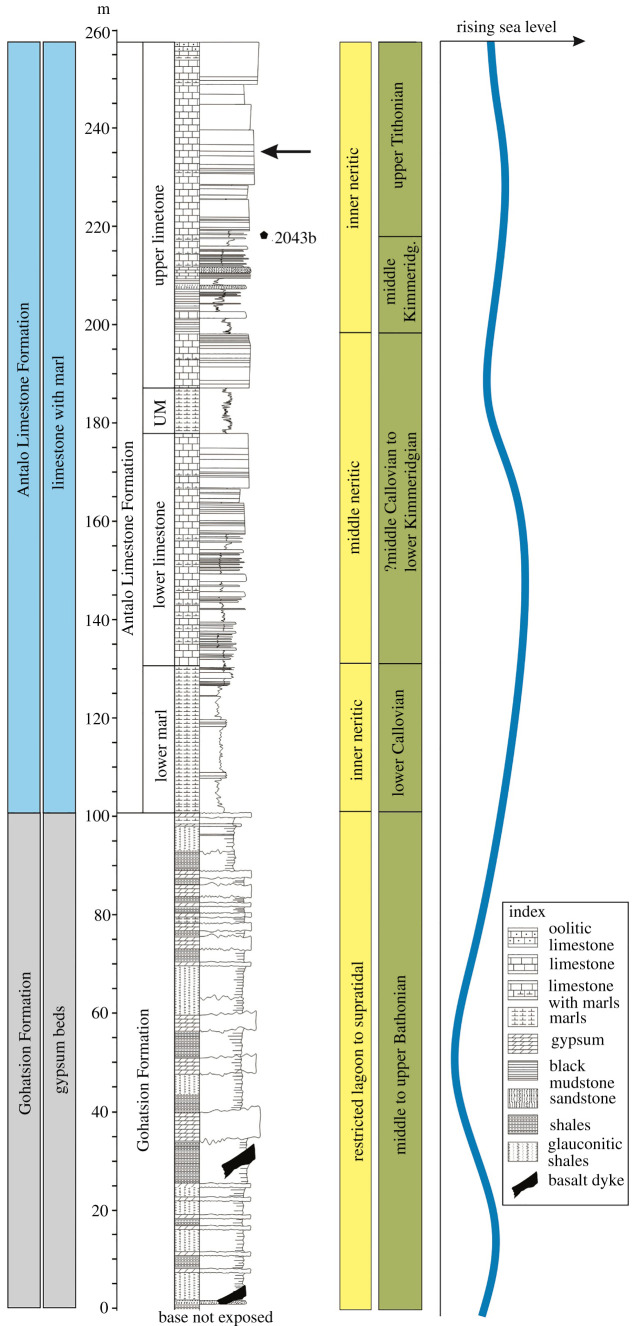
Stratigraphy of the measured section at Mugher (Blue Nile Basin; figure 1*c* for the location of the section) marking the stratigraphic position of the crinoid sample (black arrow). Additionally, the black pentagon symbol marks the position of sample 2043b that has yielded upper Tithonian calcareous nannofossils (redrawn and modified after [21]). The depositional setting and inferred relative sea-level curve for the Mugher section is redrawn and slightly modified after Jain [20] and Jain & Singh [21].

Within the Blue Nile Basin (figure 1*a*; N: 08°45′ to 10°30′; E: 36°30 to 39°00′), the Mugher section (38°24'32.0″ E; 9°31'06.9″ N) was investigated (figure 1*b,c*). The basal part of the section exposes the approximately 100 m thick Gohatsion Formation (figure 2). It comprises alternating thick units of laminated and nodular gypsum beds with chicken-wire structures with thin interbeds of glauconitic shales and yellowish siltstones (figure 2). The basalmost sediments yielded the Bathonian nautiloid, *Paracenoceras* aff. *prohexagonum* Spath [20] (figure 2). Succeeding the Gohatsion Formation, is a 160 m thick carbonate unit, the Antalo Limestone Formation (figure 2). The Antalo Limestone comprises four lithounits: (i) Lower Marl—30 m thick ash grey marls, (ii) Lower Limestone—55 m of thin-bedded ash grey limestones intercalated with ash grey marls, (iii) Upper Marls—10 m thick ash grey marls with two intervening medium-grained sandstone units, and (iv) Upper Limestone—thick-bedded pale yellow 65 m thick limestones (packstones), intercalated with pale yellow marls (figure 2). The Antalo Limestone is unconformably overlain by the coarse-grained approximately 15 m thick Mugher Muddy Sandstone, which in turn is overlain unconformably by volcanics. The Upper Limestone unit yielded the crinoid described here (38°22'32.8″ E; 9°29'03.4″ N; 2227 m elevation; figure 2). The packstone is characterized by angular and medium-sized quartz grains with small-sized broken bioclasts (mostly of bivalves) suggestive of a high-energy depositional environment. Jain & Singh [21] inferred a shoal environment for the upper parts of the Antalo Limestone Formation. The presence of nannoconids, a typical Tethyan calcareous nannofossil taxon of warm, low-latitude, carbonate shelf environments, is suggestive of warmer and nutrient-depleted surface waters (oligotrophic) [25–28]. The co-association of echinoids, gastropods and peloids are suggestive of warm shallow waters well within the upper parts of the photic zone (less than 30 m; see also [21]).

## 3. Age of the crinoid specimen

The upper part of the Antalo Limestone (the Upper Limestone; figure 2), from where the present specimen has been recorded, is dated as upper Tithonian [21]. It occurs 21 m above the calcareous nannofossil-yielding horizon that is dominated by *Nannoconus*. Globally, the occurrence of this taxa heralds the advent of the Tithonian (see also [29,30]). Additionally, this assemblage also yielded the Tithonian calcareous nannofossil marker species of *Polycostella beckmannii* and *Watznaueria communis* [21]. *Polycostella beckmannii* is the zonal marker for the NJT15b zone (upper Tithonian) and ranges up until the end of the Tithonian [31,32]. Hence, the presence and dominance of *Nannoconus* and the association of *P*. *beckmanii* suggest that the calcareous nannofossil-yielding sample 2043b is not younger than late Tithonian and that the age of the crinoid can safely be assigned as late Tithonian.

## 4. Material and methods

The comatulid specimen comes from the upper part of the Antalo Limestone Formation (38°22'49.1″ E; 9°28'41.8″ N; 2114 m elevation), 21 m above the upper Tithonian calcareous nannofossil-yielding sample 2043b [21] (figure 2).

In the neighbouring locality, Dejen (Blue Nile Basin), 150 km west of the comatulid-yielding Mugher section at a coeval upper Tithonian strata (top of the Antalo Limestone Formation (38°22'49.1″ E; 9°28'41.8″ N; 2114 m elevation; Dejen section; E0416765; N1110150; elevation 2136 m), a large stem of millericrinid (Millericrinida indet., Millericrinida) was also found. The repository of this specimen is at the Department of Geology, School of Applied Natural Science, Adama Science and Technology University, Adama (Ethiopia).

**Figure 3.**
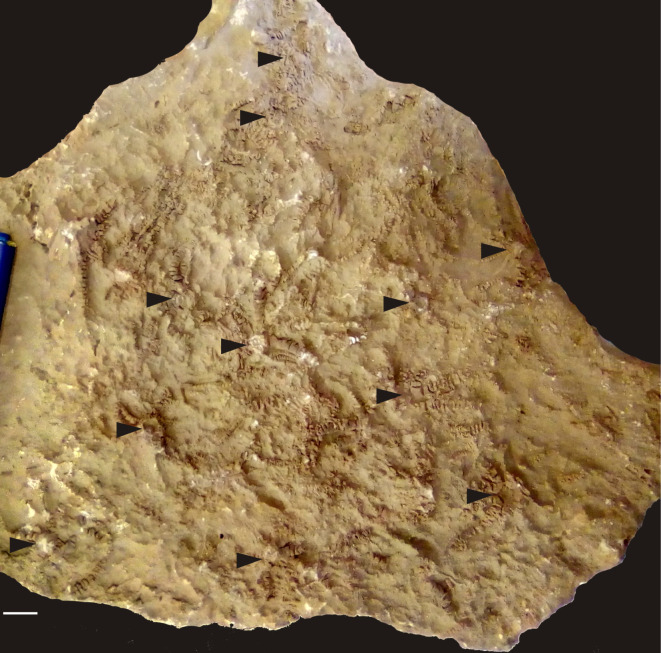
Slab showing several comatulids in upside down position (black triangulars) surrounded by putative traces of arm movements (cf. [33]). Dejen, Kurar section, Ethiopia (figure 1*c*), lower Kimmeridgian. Scale bar equals 10 mm.

During the same fieldwork, a large slab with several comatulids was also found (Dejen, Kurar section, lower Kimmeridgian) associated with the ammonite, *Orthosphinctes* aff. *tiziani* (Oppel) (see [22]) (figure 3). These specimens will be described elsewhere at a later date.

**Figure 4.**
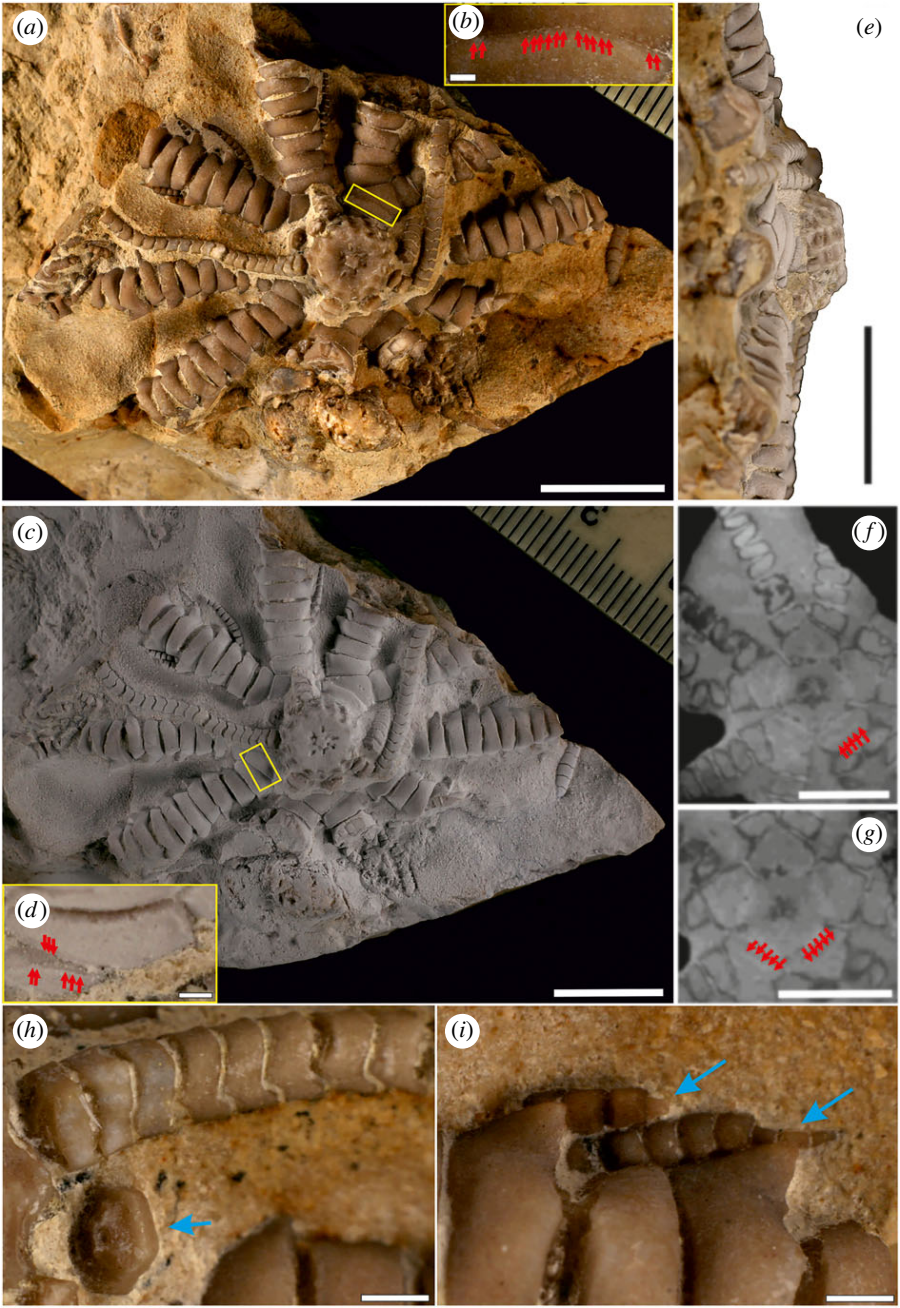
*Ausichicrinites zelenskyyi* gen. et sp. nov. from the upper part of the Antalo Limestone Formation (38°22'49.1″ E; 9°28'41.8″ N; 2114 m elevation), 21 m above the upper Tithonian calcareous nannofossil-yielding sample 2043b, Ethiopia (see also figure 2 for its stratigraphic position). Scale bar equals 10 mm (*a*,*c*,*e*,*f*,*g*) and 1 mm (*b*,*d*,*h*,*i*). (*a*,*c*). Specimen with centrodorsal, arms and cirri ((*a*) non-whitened, (*c*) whitened) with magnifications (*b*,*d*) of IBr2 articulation (note a ‘dotted’ suture line (red arrows) from the outer surface of the articulation (*b*) and a fine ridge (red arrows) on the partly exposed facet (*d*)). (*e*) Lateral view showing a centrodorsal (non-whitened). (*f*,*g*) Tomographic images of slices of the fossil comatulids showing cryptosyzygial articulation at IBr2 (red arrows). (*h*) Proximal pluricirral (lateral view) and isolated cirri (facet view, blue arrow). (*i*) Regenerating pinnules consisting of one to three pinnular plates (blue arrows).

The slab with the comatulid crinoid was collected from Ethiopia by S.J. and was transported for a detailed investigation to the Palaeontological Laboratory of the Faculty of Natural Sciences of the University of Silesia in Katowice, Poland, in September 2021. It was cleaned there and photographed using a Canon Eos 350D digital camera. This specimen was also scanned with the X-ray computed tomography (CT-scanning) using a GE Phoenix v|tome|s at the Institute of Biomedical Engineering, Faculty of Science and Technology, University of Silesia in Katowice, Poland (see electronic supplementary material, movies 1, 2). It was investigated using different settings (voltage: 140 kV, amperage: 90 A, exposure time: 333 ms, projections: 2000, voxel resolution: 5 µm). Raw two-dimensional X-ray data were then processed using Phoenix datos|x. The movies were prepared using the following application VGStudio Max 2.0. The next step was to professionally prepare the specimen using a chemical method (it was treated with potassium hydroxide—caustic potassium, KOH) by Frank Siegel, Berlin, Germany, from HAUFWERK.COM (https://www.haufwerk.com/). Finally, the specimen was photographed using a Canon Eos 350D digital camera (figure 4*a–c*).

## 5. Systematic palaeontology

**Remark.** Increasing data from molecular analyses of Recent taxa revealed that current taxonomic classifications based on morphology (e.g. [3,34,35]) are no longer tenable and thus, require substantial revision [36–39]. For instance, the morphological characters previously used to diagnose (sub)families and genera in fossil and Recent taxa (e.g. centrodorsal and cirral morphology, placement of syzygial articulations, ray branching patterns etc.) were shown to be homoplastic. Notably, morphological synapomorphies for most living genera are not available, i.e. they are usually identified by a combination of morphological features and molecular data. In this paper, given the nearly complete state of preservation of a fossil, we followed the *Artificial Key to the Families of Living Crinoids* by Charles Messing (last updated: 17 March 2021 12.20 PMURL: https://nsufl.libguides.com/crinoids). However, given the reasons above, familial placement of our fossil specimen seems uncertain (see below).

Order Comatulida A.H. Clark [40]

Suborder Comatulidina A.H. Clark [40]

Superfamily and family uncertain

*Ausichicrinites* new genus

**Type species.** *Ausichicrinites zelenskyyi* gen. et sp. nov., by monotypy.

**Zoobank.** The ZooBank life science identifiers (LSIDs) can be resolved and the associated information viewed through any standard web browser by appending the LSID to the prefix http://zoobank.org/. The LSID for this publication is: zoobank.org:pub:6A228597-F524-4021-8598-B121B21523FE, and for taxonomic registration it is: urn:lsid:zoobank.org:act:6B63762B-B4E2-4C5F-9E85-022E79872FA3.

**Etymology.** In honour of Prof. William I. Ausich for his extraordinary output to the knowledge on fossil crinoids.

**Remark.** Many features, especially of small elements, are visible using only X-ray computed tomography (see electronic supplementary material, movies 1, 2).

**Diagnosis.** Comatulid possessing 10 arms arising from five wide but low radials. Pinnules circular to oval in cross-section, without comb-like structures. Mouth central. Basals reduced to narrow rays (visible as interradial tubercles at corners of radials). Moderately low and truncated conical centrodorsal with two or three rows of cirrus sockets arranged in 15 irregular columns. Aboral end of centrodorsal cirrus-free with a distinct tubercle in the centre. IBr2 series united by cryptosyzygy. Middle and distal cirral segments smooth and longer than the proximal ones.

**Discussion.** The combination of features listed in diagnosis indicates that the fossil specimen may be assigned to the family Zygometridae (a group of Recent crinoids unknown in the fossil record). Of these features, the syzygial articulation at IBr2 is the most important familial character. However, just like in some Recent comatulids (i.e. *Zygometra microdiscus*, see fig. 17b in https://nsufl.libguides.com/crinoids), a syzygy is difficult to distinguish from a synostosis in external view in our specimen. It is somewhat more visible in X-ray computed tomography data (figure 4*f,g*), where fine depressions on the two joint faces apposing each other can be noticed. At a higher magnification, a dotted suture line from the outer surface of the articulation (figure 4*b*) and some marginal tubercles (figure 4*d*) can be observed on the partly exposed facet, which are indicative of cryptosyzygial articulation (relatively flat synostosis with short marginal radiating ridges/tubercles, cf. fig. 4 in [41]). This type of articulation is commonly present in isocrinids but is regarded as the same type of articulation as comatulid syzygy because both represent fracture points for arm autotomy (see [42]). Within Zygometridae, traditionally, two genera have been placed: *Zygometra*, in which the distal cirrus segments are much shorter than the proximal ones and bear prominent dorsal spines, and *Catoptometra*, without dorsal spines. However, recent molecular analyses noted Zygometridae as polyphyletic [43], hinting that the syzygy at br1 + 2 lacks the familial taxonomic importance [40], although this feature still appears characteristic for these two genera. Notwithstanding, our specimen differs from those two Recent genera by having distal cirrus segments smooth and much longer than the proximal ones. The morphology of centrodorsals of the specimen at hand is also consistent with Zygometridae. Representatives of this family may possess centrodorsals displaying highly varied morphologies even within a single species ([15], p. 99: ‘…from large to flattened himisperical to small and rather thin discoidal, being usually large and thick discoidal with the dorsal pole slightly concave or almost flat… The cirrus sockets are arranged in 2 or 3 rows, sometimes more, crowded and irregular marginal rows’).

On the other hand, the centrodorsal morphology of *Ausichicrinites* is also similar to those observed in the family Solanocrinitidae Jaekel, widely represented in the Upper Jurassic, which may possess truncated conical controdorsals with cirrus-free rugose or flat aboral apex. However, the centrodorsal of *Ausichicrinites* appears lower and in its central aboral part there is a strongly raised tubercle. According to Hess and Thuy [44], family Solanocrinitidae contains four genera: *Solanocrinites*, *Archaeometra*, *Comatulina* and *Pachyantedon*. Rasmussen [45], Hess and Messing [3] and Hess [9] emphasized that, on one hand, solanocrinitid taxa are characterized by a conspicuous variation, but on the other hand, they may be very similar. This note especially applies to *Solanocrinites*, *Archaeometra* and *Comatulina*. Hess & Messing [3] stated that centrodorsal of *Solanocrinites* is very similar to *Archaeometra*. They added that the adoral diameter of the *Archaeometra* is approximately twice that of the aboral apex. In our view, this is only true for adults. In the case of juveniles, the adoral diameter is only slightly larger than that measured in the aboral apex (cf. fig. 4a versus 4c in [46]). Hence, in our opinion, there is no way to distinguish juvenile specimens of these two taxa. Hess [9] also pointed out that in *Solanocrinites* and the closely related *Comatulina*, the cirrus sockets occur in regular columns separated by vertical ridges, and the basal circlet is tightly joined to the radial circlet. *Pachyantedon* was interpreted by Jaekel [47] as a 10-armed comatulid. In our opinion, this form, as proposed by Rasmussen [48], should be treated as indeterminable crinoid due to the fact that it is preserved in a piece of flint, its centrodorsal is invisible, and the specimen has only 10 biserial arms, and two cirral fragments. Moreover, the age of this specimen is considered ‘presumably’ Late Cretaceous (e.g. [3,48]). Although the general shape of the centrodorsal in *Ausichicrinites*, as mentioned above, resembles to some extent that observed in *Solanocrinites*, there are a number of features that differentiate both taxa. In particular, in solanocrinitids the first and the second primibrachials are fused, or rarely joined by synostosis, whereas secundibrachials are strongly wedge-shaped and commonly spinose (fig. 39 in [3]). Furthermore, solanocrinitids commonly have more exposed stout rod-shaped basals and overhanging radials, distinctly higher than in *Ausichicrinites* (fig. 38a in [3]).

*Ausichicrinites zelenskyyi* new genus and species.

Figure 4*a–i*, electronic supplementary material, movies 1, 2.

**Type material.** The holotype is a nearly complete specimen (without median-distal arm parts and some cirrals preserved) stored at the Department of Geology, School of Applied Natural Science, Adama Science and Technology University, Adama (Ethiopia) with the specimen numbered ASTU/Geol-SJ/2018/2-1. The specimen is from the upper part of the Antalo Limestone Formation (38°22'49.1″ E; 9°28'41.8″ N; 2114 m elevation), 21 m above the upper Tithonian calcareous nannofossil-yielding sample 2043b [21] (figure 2).

**Measurements.** Centrodorsal diameter in basal part: 7.91 mm; centrodorsal diameter in proximal part: 8.23 mm; centrodorsal height: 4.70 mm; cirrus scar sockets diameter: 1.10–1.29 mm; arm thickness: 3.20–5.20 mm; cirri diameter: 0.58–1.89 mm; pinnules diameter: 0.29–0.78 mm.

**Etymology.** In honour of Volodymyr Oleksandrovych Zelenskyy, the sixth and current president of Ukraine for his courage and bravery in defending free Ukraine.

**Diagnosis.** As for the type species by monotypy.

**Description.** Ten massive and uniserial arms. Secundibrachials tumid, wedge-shaped and united by muscular (and sometimes symmorphial) synarthry. Pinnules circular to oval in cross-section without comb-like structures; P1 from br2-br4. Rays divided at primibrachial 2. IBr2 series united by poorly visible cryptosyzygial articulation. Mouth central. Five short but wide radials. Basals reduced to narrow rays (visible as interradial tubercles at corners of radials). Centrodorsal moderately low and truncated conical, slightly five-sided. Aboral end of centrodorsal concave, cirrus-free, and with a distinct tubercle in the centre; the axial pore is narrow. Cirri are arranged in two or three rather irregular rows; cirral sockets are closely placed, of different size, rather deep, oval to hexagonal and arranged in 15 columns; in total of 35 sockets on centrodorsal. Middle and distal cirrus segments smooth and longer than the proximal ones. Cirri more than XIX.

**Stratigraphic distribution.** Upper Tithonian of Ethiopia.

## 6. Fossil comatulids—an overview

According to Hess and Messing [3] (see also [45,48] and references therein; [7,49]) comatulids are represented by 189 genera (excluding the suborder Bourgueticrinina Sieverts-Doreck that was assigned by Hess & Messing [3] to the order Comatulida), of which 39 are known from the fossil record (excluding taxa whose stratigraphic range also includes the Holocene). Of these, only a few are documented based on articulated centrodorsals with basals/radials, arms and cirri (sometimes with pinnules).

### 6.1. Mesozoic

The oldest and nearly complete specimens assigned to ‘protocomatulids’ are representatives of the genus *Paracomatula* (i.e. *Paracomatula triadica* Hagdorn and Cambell known from the Upper Triassic of New Caledonia; figs. 3–6 in [10]). However, it should be noted that the paracomatulids are generally classified under Pentacrinitina Gray (suborder of Isocrinida Sieverts-Doreck). These crinoids are characterized by the presence of moderately massive, 10 arms, divided once at primibrachial 2. Between primibrachials 1 and 2 and secundibrachials 1 and 2, synarthries are visible; cryptosyzygy with fine ridges occurs between secundibrachials 3 and 4. After secundibrachials 11 or 12, syzygies occur on approximately every fifth brachial. Pinnules are delicate, and the first pinnule is present on secundibrachials 2.

Among the most spectacular finds of ‘true’ comatulids are representatives of the Late Jurassic *Solanicrinites* (Solanocrinitidae) collected from a locality in the vicinity of Basel (Switzerland) (e.g. fig. 39 in [3], T79). Other finds of this genus are known from Germany, UK, France and Poland (e.g, [3,45,48,50–53]). Their brief morphologic description is provided above (see Discussion in the Systematic palaeontology section).

Other finds of ‘true’ comatulids comprising centrodorsals with preserved arm fragments, and sometimes cirri, belong to the family Pterocomidae Rasmussen. One of the most complete comatulid of this family is *Comaturella pennata* von Schlotheim from the Tithonian lithographic shales of Germany (see p. 326 in [54]). It possesses 10, extremely long and thin arms, divided at primibrachial 2. First primibrachials are low and wide. Between secundibrachials 3 and 4 syzygy occurs. Pinnules covering arms are long, up to 15 mm, with 15 to 20 pinnulars. They are slender and swollen at articulations. Distal pinnules are as long as proximal ones. Cirrals are slender and without spines. Distal cirrals are long, distinctly longer than wide. Another representative of this family is the 10-armed *Rhodanometra lorioli* Manni, Nicosia and Riou from the Callovian of France ([55], p. 88, fig. 4). Its arms are moderately long and massive. Pinnules do not possess a longitudinal ridge or crest, and the proximal brachials lack pinnules. Its cirri consist of 19 cirrals. Proximal cirrals are short, whereas the distal ones are long and slender.

Another nearly complete specimen, of uncertain family, belongs to the genus *Procomaster* (i.e. *Procomaster pentadactylus* Simms from the Toarcian of Germany). This form has five arms comprising extremely low brachials throughout. The synarthries occur between primibrachials 1 and 2, and syzygies are present at approximately every third to fifth articulation. This form also possesses 25 robust and strongly recurved cirri. Cirrals are rounded rhomboidal with the fulcral ridge parallel to the minor axis ([56], figs. 1–4).

Somewhat less completely preserved comtaulids (centrodorsal plus small arm parts) include: (i) *Palaeocomaster calloviensis* Gislén (Solanocrinitidae) from the Callovian of England ([57], pl. 1) with synarthries between primibrachials 1 and 2 and secundibrachials 1 and 2, and syzygies with few ridges between secundibrachials 3 and 4; (ii) *Pseudoantedon hiselyi* (de Loriol) (Decameridae) from the Hauterivian of Switzerland ([58], pl. 12) with five massive arms; its first pinnules are not carinated and occur on first brachial, and its cirrals are rounded and lack aboral spines; (iii) *Glenotremites loveni* (Carpenter) (Notocrinidae) from the upper Albian of France (pl. 5 in [59]; see also figs. 19 and 20 in [45]) with 10 arms and with syzygies between secundibrachials 3 and 4, 9, 10 and distally at interval of 5 ossicles; (iv) *Antedon* (=*Semiometra*) *impressa* (Carpenter) (Notocrinidae) from the Bathonian of England ([59], pl. 4); its brachials are known from one putative specimen [3], and its first pinnule occurs on secundibrachial 2; (v) *Hertha mystica* von Hagenow (Antedonidae) from Denmark, Germany and Sweden [45]; its arms are divided at primibrachial 2, and there are synarthries between primibrachials 1 and 2; (vi) *Roiometra columbiana* A.H. Clark (Antedonidae) is from the Albian of Colombia; it is characterized by a centrodorsal with 10 arms, preserved cirrals, and pinnulars; however, there is no suitable description and illustration for this species ([60], p. 304); (vii) *Decameros ricordeanus* (Decameridae) with five arms has no syzygies or synarthries, and the first pinnule occurs on the first brachial. It is noteworthy that Saulsbury and Zamora [61] recently described a well-preserved representative of this species that preserved the shape and configuration of the nervous and coelomic canals.

### 6.2. Palaeogene and Neogene

Some limited morphologic data on the arms of *Discometra* (Himerometridae), *Bruennichometra* (Conometridae), *Palaeantedon* (Antedonidae) and *Microcrinus* (superfamily and family uncertain) are known; but they mostly come from disassociated ossicles that were not attached to centrodorsals. The arm plates of *Discometra* recorded from Africa (Algeria) and Europe (Austria, France, Germany, Hungary, Italy) are represented by typical syzygial brachials. Noteworthy cirrals of this taxon are also known; they are smooth and lack aboral spines [3,14,62,63]. Brachials of *Bruennichometra* recorded from Denmark [45] are smooth or granular, and most often with a median crest. Synarthry is observed between primibrachials 1 and 2. All adjacent first primibrachials are jointed laterally. Distal brachials are with muscular and syzygial articulations [45]. *Palaeantedon*, known from Africa (Algeria), Europe (Italy, Hungary) and North America (USA), has a synarthry between primibrachials 1 and 2, and the second primibrachial is axillary [14,63,64]. Isolated brachials of *Microcrinus* from North America (USA) indicate that its arms were divided at the primibrachial 2. The primibrachial 1 is rather high. Synarthy occurs between primibrachial 1 and 2 and secundibrachials 1 and 2. Other brachial contacts are syzygial [3,65,66].

Eagle [67] created two new genera: *Maorimetra* with *M*. *ardlogiensis* as type species and *Zelandimetra* with *Z. neozelandiae* from Oligocene of New Zealand. These forms are only based on centrodorsals. However, these individuals should probably be associated with one of the representatives of *Asterometra* or *Pterometra*. Also, Hess & Messing [3], in the revised *Treatise*, did not include them under the Comatulida.

The most complete post-Mesozoic feather stars, excluding modern taxa with a stratigraphic range beyond the Holocene, are: *Kiimetra miocenica* Shibata and Oji from the Miocene of Japan [68] and *Rautangaroa aotearoa* (Eagle) from the Oligocene of New Zealand [49]. The centrodorsal of *K. miocenica* is hemispherical with a dorsal star with 10 to 20 slender arms. The aboral surface of brachials is smooth and devoid of carination. Its pinnules are stiff and triangular in outline, and cirri consist of at least 40 squarish cirrals. *Rautangaroa aotearoa*, in turn, has a poorly preserved large, pentastellate, truncated conical centrodorsal and at least 21 arms, divided at primibrachial 2 and secundibrachial 2. All brachials are smooth and wider than high. The cirri are disarticulated, cylindrical to long, compressed and hour-glass shaped (for summary table 1).

**Table 1.** List of Mesozoic-Neogene comatulid genera (excluding those with a stratigraphic range beyond the Holocene; compiled after [3,7,9,14,44,45,48,49,52,55,56,68–70]).

genus	type species	range	characters of centrodorsal	characters of cirri/cirrals	characters of arms/brachials	reference
*Paracomatula*	*P. helvetica*	Upper Triassic (Norian)–Middle Jurassic (Bajocian)	centrodorsal low, composed of united, articulated, 5-sided columnals; cirrus sockets without profile, arranged in 5 or 10 columns	cirri long, all cirrals are smooth; distal and middle cirrals are longer than proximal ones	moderately massive, 10 arms; rays divided once at primibrachial 2; between primibrachials 1 and 2 and secundibrachials 1 and 2 synarthry; cryptosyzygy with fine ridges occurs between secundibrachials 3 and 4; after secundibrachials 11 or 12 syzygies occur on approximately every fifth brachial	[8]
*Palaeocomaster*	*Actinometra guirandi*	Lower Jurassic (Hettangian)–Upper Jurassic (Portlandian)	centrodorsal low discoidal; cirrus sockets not forming columns, irregular in 1 to 3 circles	—	synarthries between primibrachials 1 and 2 and secundibrachials 1 and 2; syzygies with few ridges between secundibrachials 3 and 4	[14]
*Forcipicrinus*^a^	*F. normannicus*	Lower Jurassic (Toarcian)	centrodorsal columnal moderately thick; cirrus sockets deeply sunken	—	—	[44]
*Spinimetra*^a^	*S. chesnieri*	Lower Jurassic (Toarcian)	centrodorsal circular and cone shaped in the adult specimens; hemispherical in the juvenile specimens; cirrus sockets numerous and small, crowded and sunken, with pronounced horseshoe-shaped rim	—	—	[44]
*Procomaster*	*P. pentadactylus*	Lower Jurassic (Toarcian)	centrodorsal small and slender? concealed by the cirri	25 robust and strongly recurved cirri; cirrals rounded rhomboidal with the fulcral bar parallel to the minor axis	five, composed throughout of extremely low brachials; synarthries between primibrachials 1 and 2, and syzygies at approximately every third to fifth articulation	[56]
*Andymetra*^a^	*A. galei*	Lower Jurassic (Toarcian)–Middle Jurassic (Bathonian)	centrodorsal hemispherical, apex with only small cirrus-free area; cirrus sockets crowded, deep and hardly sculptured and occur in several, irregular rows	—	—	[70]
*Singillatimetra*^a^	*S. inordinata*	Middle Jurassic (Bathonian)	centrodorsal low, asymmetrical in outline; 5 bulging cirrus sockets arranged irregularly	—	—	[70]
*Rhodanometra*	*R. lorioli*	Middle Jurassic (Callovian)	centrodorsal subpentagonal and convex; cirrus sockets smooth, 2 or 3 in radial areas	—	10 moderately long and massive; no pinnules on proximal brachials	[55]
*Solanocrinites*	*S. costatus*	Middle Jurassic–Upper Jurassic (Tithonian)	centrodorsal moderately high discoidal or truncated conical, more or less 5-sided; cirrus sockets in 10 columns, from 1 to 3	cirri are very long, all cirrals are smooth; distal and middle cirrals are longer than proximal ones	massive arms, which may be uni- or bi-serial; first and second primibrachials fused, or sometimes, joined by synostosis; rays divided at primibrachial 1 in fused primibrachials or rarely at primibrachial 2; secundibrachials wedge shaped and tumid, often spinose	[52]
*Archaeometra*	*Solanocrinites asper*	Middle Jurassic (Bajocian)-Lower Cretaceous (Valanginian)	centrodorsal high discoidal or truncated conical, 5-sided; cirrus sockets in 10 columns, from 1 to 3	—	synarthry or cryptosynarthry between primibrachials 1 and 2; primibrachial 2 axillary; syzygies with stout ridges	[71]
*Semiometra*	*Antedon impressa*	Middle Jurassic (Bathonian)–Palaeogene (Eocene)	centrodorsal low, disk shaped with a dorsal star; cirrus sockets crowded, very small, regularly alternating but not forming columns	—	first pinnule on secundubrachial 2	[14]
*Comatulina*^a^	*C. costata*	Middle Jurassic (Callovian?), Upper Jurassic (Oxfordian)–Upper Cretaceous (Coniacian)	centrodorsal truncated conical to truncated subhemispherical; cirrus sockets closely placed in 1 to 3 columns	—	—	[51]
*Hrabalicrinus*^a^	*H. zitti*	Upper Jurassic (Oxfordian)	centrodorsal high, truncated, distinctly 5-sided; cirrus sockets large, in 10 columns, 2 to 3 sockets per column large	—	—	[7]
*Comaturella*	*Ophiurites pennatus*	Upper Jurassic (Tithonian)–Upper Cretaceous (Turonian)	centrodorsal low, discoidal; 10 cirrus sockets in a single marginal row	cirrals slender and without spines; distal cirrals long, distinctly longer than wide	10 extremely long and thin arms; rays divided at primibrachial 2; first primibrachials low and wide; between secundibrachials 3 and 4 syzygy occur; pinnules long, with 15 to 20 pinnulars.	[72]
*Pseudoantedon*	*P. icauensis*	Lower Cretaceous (Berriasian?, Valanginian–Barremian)	centrodorsal small, discoidal; aboral apex flattened; adoral side with coelomic furrows	cirrals rounded without aboral spines	five, massive arms; rays undivided; first pinnules occur on first brachial	[73]
*Coelometra*	*Antedon campichei*	Lower Cretaceous (Valanginian)	centrodorsal high, truncated subconical to hemispherical; cirrus sockets large, incompletely covering centrodorsal	—	primibrachial 1 axillary; articulations between secundibrachials 1 and 2 oblique muscular	[74]
*Decameros*	*D. ricordeanus*	Lower Cretaceous (Valanginian–Albian)	centrodorsal large, discoidal; cirrus sockets from small to large, more or less circular, irregularly placed	—	five arms with no syzygies or synarthries, and first pinnule on the first brachial	[51]
*Roiometra*	*R. columbiana*	Lower Cretaceous (Albian)	centrodorsal hemispherical or subconical; cirrus sockets numerous (even more than 100) in several alternating circles	—	10 arms	[75]
*Remesimetra*^a^	*Glenotremites discoidalis*	Lower Cretaceous (Albian)–Upper Cretaceous (Turonian)	centrodorsal rounded subconical to discoidal; cirrus sockets large with articular tubercles and marginal crenulae in 20 irregular columns	—	—	[14]
*Glenotremites*	*G. paradoxus*	Lower Cretaceous (Albian)–Upper Cretaceous (Santonian)	centrodorsal hemispherical to discoidal with a dorsal star; cirrus sockets large, irregularly crowded, with lateral tubercles and marginal crenulae	—	10 arms with syzygies between secundibrachials 3 and 4, 9, 10, and distally at interval of 5 ossicles	[50]
*Pachyantedon*	*P. beyrichi*	Upper Cretaceous	?	cirri stout?	10 arms composed of wedge-shaped brachials	[47]
*Amphorometra*^a^	*Glenotremites conoideus*	Upper Cretaceous (Cenomanian)–Palaeogene (Danian)	centrodorsal conical to slightly truncated conical; cirrus sockets with narrow canal and indistinct fulcral ridge placed in 10 columns	?proximal cirrals short and smooth, wider than long	—	[50]
*Placometra*^a^	*P. mortenseni*	Upper Cretaceous (Turonian)–Palaeogene (Danian)	centrodorsal high conical or truncated conical; cirrus sockets very few, large and high, elliptical, from 1 to 3 per column	—		[14]
*Schlueterometra*^a^	*S. voigti*	Upper Cretaceous (Coniacian–Santonian)	centrodorsal conical; cirrus sockets from small to large in 10 columns	—	—	[45]
*Loriolometra*^a^	*Comaster retzii*	Upper Cretaceous (Campanian)	centrodorsal large and high, slightly conical; cirrus sockets large, arranged in 10 columns	—	—	[76]
*Jaekelometra*^a^	*Atelecrinus belgicus*	Upper Cretaceous (Campanian)–Palaeogene (Danian)	centrodorsal low to high convex conical; cirrus sockets in 10 columns, with more or less distinct fulcral ridge or tuberlces	—	—	[77]
*Hertha*	*H. mystica*	Upper Cretaceous (Maastrichtian)–Miocene	centrodorsal arched, high, subconical to low discoidal; cirrus sockets small and crowded	—	rays divided at primibrachial 2; synarthries between primibrachials 1 and 2	[78]
*Atuatucametra*^a^	*A. annae*	Palaeogene (Danian)	centrodorsal low to slightly conical; cirrus sockets small, irregularly arranged	—	—	[79]
*Bruennichometra*	*Antedon danica*	Palaeogene (Palaeocene)	centrodorsal truncated conical to hemispherical; cirrus sockets large with distinct fulcral ridge arranged from 1 to 3 and placed in 10 columns	—	proximal brachials smooth or granular, most often with a median crest; synarthry between primibrachials 1 and 2; all adjacent first primibrachials jointed laterally; distal brachials with muscular and syzygial articulations	[45]
*Vicetiametra*^a^	*V. albertinii*	Palaeogene (Eocene)	centrodorsal hemispherical to truncated subconical; cirrus sockets large, irregularly arranged, more or less 15 columns from 1 to 2 sockets	—	—	[80]
*Microcrinus*	*M. conoideus*	Palaeogene (Eocene)	centrodorsal pentagonal, conical; cirrus sockets circular, moderately large, placed in 10 columns	—	rays divided at primibrachial 2; primibrachial 1 high; synnarthy between primibrachial 1 and 2 and secundibrachials 1 and 2; other conntacts are syzygial	[65]
*Conometra*^a^	*Alecto alticeps*	Palaeogene (Eocene)–Neogene (Miocene)	centrodorsal conical to truncated conical; cirrus sockets with indistinct fulcral ridge from 4 to 5 in 15 columns	—	—	[14]
*Palaeantedon*	*Antedon solutus*	Palaeogene (Eocene)–Pleistocene	centrodorsal arched to hemispherical; cirrus sockets small and numerous, closely placed	—	synarthry between primibrachials 1 and 2; second primibrachial axillary	[14]
*Rautangaroa*	*Cypelometra aotearoa*	Palaeogene (Oligocene)	centrodorsal large, pentastellate, truncated conical; cirrus sockets concave, moderately deep, and covered with radiating crenulae along the margins	cylindrical to long and compressed, hour-glass shaped	at least 21 arms; arms divided at primibrachial 2 and secundibrachial 2; brachials smooth, wider than high	[49]
*Moanametra*^a^	*M. torehinaensis*	Palaeogene (Oligocene)	centrodorsal arched conical; cirrus sockets in 15 columns from 2 to 4	—	—	[81]
*Cypelometra*^a^	*Antedon iheringi*	Neogene (Miocene)	centrodorsal hemispherical to subconical; cirrus sockets in 10 columns from 3 to 4	—	—	[14]
*Discometra*	*Eugeniacrinus*? *rhodanicus*	Neogene (Miocene)	centrodorsal low hemispherical; cirrus sockets closely placed in 3 to 5 tiers	—	syzygies in some brachials	[14]
*Kiimetra*	*K. miocenica*	Neogene (Miocene)	centrodorsal hemispherical with dorsal star; cirrus sockets arranged in 2 or 3 irregular rows		10 to 20 slender arms, aboral surface of brachials smooth and devoid of carination; pinnules stiff and triangular in outline	[68]
*Allionia*^a^	*A. oblitia*	Neogene (Miocene)	centrodorsal low, pentagonal; cirrus sockets small, closely placed	—	—	[82]

^a^Taxa exclusively known from centrodorsal accompanied by basals and/or radials.

Other spectacular finds include comatulids form the Eocene La Meseta Formation of Seymour Island, Antarctic Peninsula (*Notocrinus rasmusseni* Meyer and Oji, and *Notocrinus seymourensis* Baumiller & Gaździcki [83]). They have bowl-shaped centrodorsals with cirri sometimes bearing a keel. Articulation between first and second primibrachials are synarthrial, and distal syzygies are present at interval of 2 to 20. Recent representatives of this genus have gonads at base of genital pinnules.

### 6.3. Regeneration signs

Signs of arm regeneration are commonly documented in fossil stalked crinoids (e.g. [84,85]); however, this phenomenon has been rarely documented in fossil comatulids probably due to the fact that intact specimens of these crinoids are exceedingly rarely preserved. To date, only a single report has been described, namely a regenerating arm consisting of four tetribrachs in *Rautangaroa aotearoa* from the Oligocene of New Zeland [49]. Two pinnules of *Ausichicrinites* bear clear signs of regeneration (figure 4*i*). It is clearly recognized by abrupt differences in the size of abutting pinnular plates. Thus, new fossil evidence from Africa constitutes the earliest and the first example of pinnule regeneration in a fossil comatulid. This finding supports the hypothesis about crucial role of predation in the evolutionary history of this group [4,5]. In the Recent environments, numerous examples of ‘pinnule predation’ by fishes have been reported (e.g. [86]). It has been argued that such interaction may represent the so-called 'collateral damage’ [87], i.e. the attacks by fishes are focused on commensals living in the crinoid arms.

## 7. Concluding remarks

The only Mesozoic comatulid found in Africa, *Amphorometra bellilensis* (Valette) from the Cenomanian or Turonian in the Djebel Bellil from south Tunisia, is based on a single centrodorsal ([88], p. 374, fig. 3). The described specimen (*Ausichicrinites zelenskyyi* gen. et sp. nov.) represents one of the most complete fossil comatulids known to date and enables unique insights into the morphology of its arms and cirri. This record is also the oldest one from the African continent. Given the high post-mortem disarticulation gradient in comatulids, it is likely that the described specimen was buried rapidly, possibly when still alive. Distal arm parts and some cirri might have been lost during storm disturbance (high energy, shallow water conditions) and/or after the specimen was exposed on the outcrop. The described taxon has a combination of characters not observed in any of the fossil comatulids, and rarely noted in Recent ones. Its pinnules and cirri are slender, without any spines or comb-like structures, a character typically observed in fossil comatulids. By contrast, pinnules and cirri of Recent forms commonly display some ornamentation. Most notably, IBr2 series in *Ausichicrinites* are united by poorly visible cryptosyzygy. This feature is only observed in two genera of living crinoids, i.e. Zygometra and *Catoptometra*, that are classified within Zygometridae, currently considered as a polyphyletic family only known from the Holocene of western Pacific and eastern Indian Oceans. This may suggest that the evolutionary origins of the family need to be pushed back by *ca* 150 million years. This scenario is, however, highly unlikely given the commonly occurring homoplasy (morphological convergences) in crinoids [87]. Rather, our fossil illustrates high phenotypic convergences among crinoids. The described specimen shows evidence of pinnule regeneration, which constitutes the first example of this phenomenon in a fossil feather star.

## Data Availability

The unprocessed raw data from microCT tomography of *Ausichicrinites zelenskyyi* are available from the Dryad Digital Repository: https://datadryad.org/stash/share/3NcQHS9sfYHe5GkxwesbMuyyubklYGdZY_0khnygz3c. Electronic supplementary material is available online [89].
